# Implementing Prevention against Tobacco Dependence (PAD) "Toward the Tobacco-Free Schools, Neighborhoods, and Cities": Study Protocol

**DOI:** 10.34172/jrhs.2020.23

**Published:** 2020-08-24

**Authors:** Mohammadreza Masjedi, Sonia Ghaffari, Payam Roshanfekr, Mohammadreza Bahrami Hessari, Sanaz Hamzehali, Abzar Ashtari Mehrjardi, Elham Moaaf, Hamidreza Shahsavan

**Affiliations:** ^1^Tobacco Control Research Center, Iranian Anti-Tobacco Association, Tehran, Iran; ^2^Social Welfare Management Research Center, University of Social Welfare and Rehabilitation Sciences, Tehran, Iran

**Keywords:** Tobacco, Smoking Prevention, Schools, Public Health, Primary Prevention, Urban Health

## Abstract

**Background:** Although tobacco consumption in Iran has decreased in recent years, in 2010, the exposure to cigarette smoke was the fifth leading risk factor for death in Iran. This article is presenting the protocol for the prevention against tobacco dependence (PAD) project, an initiative planned and implemented by the Iranian Anti-Tobacco Association (IATA) of Iran in the city of Varamin.

**Study design:** A prospective cohort study.

**Methods:** This project is carried out based on a participatory community-oriented approach and an action research method. It includes four inter-related, prospective studies phases; pilot, tobacco-free school (TFS), tobacco-free neighborhood (TFN), and tobacco-free city (TFS). The measuring tools for each phase were designed primarily using CDC and WHO guidelines and preliminary details were identified. Each phase is a combination of different methods (including systematic observation, questionnaire, heuristic interview, and structured interview). The studies will examine twelve goals and meet 9 project objectives in a comprehensive evaluation of ongoing progress with TFS, TFN, and TFC.

**Discussion:** This project seeks to achieve indicators of tobacco-free schools, neighborhoods, and cities through direct and indirect education of all the target groups in the community. Participation of stakeholders and supporters in problem-solving can increase the effectiveness and influence of the project. The outcomes of the first two phases will be expanded to the wider settings.

## Introduction

Every year more than 8 million people die because of consumption of tobacco (>7 million) or exposure to second-hand smoke (~1.2 million)^1^. Many of the chemical substances found in tobacco smoke are hazardous to health and 69 of them are known to be carcinogens ^2^. Half of the children have exposure to second-hand smoke in public places that causes 65000 death annually^1^.

According to a study in 2010, the exposure to cigarette smoke was the fifth leading risk factor for death in Iran ^3^. In the latest WHO report on the global tobacco epidemic for Iran, the prevalence of any tobacco use in 2017 for those aged 15 year or more was 25.1 for men and 3.6 for women^4^.

A large study of about 3,500 smokers in six provinces of Iran showed that most of them (67%) had started smoking from the age of 10 to 19 years ^5^. Prevention at younger ages and in educational settings is emphasized ^6^. Researchers have warned the increase in the prevalence of smoking among adolescents and young people in Iran^7^. In a study with the participation of 4.591 students aged 17 to 19 year, 12.1% of boys and 5.3% of girls had a history of smoking^8^. In another large scale study, 2.6% of students self-reported that they were smokers (3.5% of boys and 1.7% of girls) and also 5.6% of the students had a history of smoking (7.5% of boys and 4.2% of girls). The prevalence of smoking in people aged 14 and 18 year (6.11%) was higher than those aged 10 to 14 years (1.18%)^9^. In all socio-economic groups, one of the predictors of the tendency to smoking was having a smoking father ^10^. According to the STEPS study in 2016, 14.82% of Iranians aged 18 to 24 year had used hookah ^11^.

Various activities have been undertaken to prevent tobacco use in Iran^12^. In 2003, Iran signed the WHO Framework Convention on Tobacco Control (FCTC). The various items of FCTC have been taken into account in policies and plans. The prevalence of tobacco use has not increased in the past two decades, but the burden of diseases is still at a high level and more effective preventive strategies are needed ^13^.

The participation of adolescents and young people in tobacco prevention programs improving the effectiveness of them ^14^, and comprehensive community-wide interventions such as campaigns that engage the families help with the effectiveness of school-based interventions^15^. Besides, the intervention of these school-based programs should consider the norms of society ^16^.

Due to the lack of effective and participatory programs which consider prevention of smoking from the younger ages, this study is aimed at preventing tobacco use (cigarettes, hookahs, and chewing tobacco) among adolescents (tobacco-free schools), also discusses achieving tobacco-free neighborhoods and cities through the participation of adolescents.

In this article, the framework for the phases and the protocol for continuing this project are presented.

## Methods

This project is to be implemented for 4 years in the city of Varamin which is one of the southern cities of Tehran Province, Iran with about 300,000 population and 85,000 households, with 18% of its population as students. The literacy rate in this city is about 87%, which is 1% less than the average literacy rate of the whole country but ranked 13^th^ among the 14 counties of Tehran Province. The rate of economic participation of Varamin is lower than the rate of economic participation in the whole country (35.62 out of 100 in Varamin, compared to 39.4 in the whole country) ^17^. Varamin has a high ethnic and cultural diversity and there are a large number of domestic and foreign (Afghans) immigrants in it.

After conducting initial observations and confirming the problems caused by tobacco use in Varamin school students, the Tobacco Control Research Center, affiliated with the Iranian Anti-Tobacco Association, planned a participatory research project in the city’s schools due to its special location. Varamin, as one of the cities near Tehran metropolis, faces various social harms. Due to having a largely immigrant population, both from inside Iran and from neighboring countries, this city is not only faced with ethnic diversity but also with a variety of habits regarding the consumption of tobacco products.

After attracting the cooperation of the Education Department of Varamin City and school principals, in order to recognize the existing challenges and to get a more accurate understanding of the literature on the subject and the field of research, field studies, and heuristic activities began.

In preliminary studies, global experience with school-based interventions has shown that interventions may be weak or even ineffective if the environment in which the school is located is neglected. For example, in guidelines for tobacco prevention programs in schools ^18^ and according to previous research on the subject^19^, CDC reports that to improve the effectiveness of school-based programs, the participation of parents, mass media, especially which can affect youth, and community organizations is necessary.

Finally, after reviewing the literature, interviews with key informants in Varamin, and participation of related institutions (such as Ministry of Education and the Municipality), as well as to make the project more effective and have a comprehensive perspective, the neighborhood and city phases were added to the next phases of the research.

This project has been designed in four phases, and with the following targets:

### Phase 1: Pilot project

Targets:

- Possibility assessment of the (pilot) implementation of the project in selected schools in the city;
- Measuring the effectiveness of interventions.

### Phase 2: Tobacco-free school

Targets:

- Generalization of the pilot phase to all schools;
- Achieving the indicators of the tobacco-free school.

### Phase 3: Tobacco-free neighborhood

Targets:

- Possibility assessment of the (pilot) development of the project in selected neighborhoods of the city;
- Achieving the indicators of the tobacco-free neighborhood.

### Phase 4: Tobacco-free city

Targets:

- Generalization of the pilot implementation of the third phase to all the neighborhoods of Varamin City and pursuing the implementation of the anti-tobacco laws and instructions in public places;
- Achieving the indicators of the tobacco-free city.

This project is carried out based on a participatory community-oriented approach and an action research method. Action research is defined as a method for interacting with a system (i.e. community) with two goals of learning from it and making changes to it^20^, so it can also be called collaborative research ^21^. In this way, action research can be considered as a community-based approach in which the role of the researcher is to facilitate changes and to evaluate them^22^.

### Sampling method and sample size calculations

For the first phase, the statistical population is all the seventh-grade students in Varamin City. Using cluster sampling and Cochran’s formula, 920 students will be selected out of 64 schools (with two variables of gender and type of school [public or private]), as the sample size for the first phase.

In the next phases, there will be no sampling as the target groups are all the students over 10 year old in the city, all the residents of the selected neighborhoods, and all the residents of the city.

### Measuring intervention

For the assessment of the intervention in the pilot phase, we will employ the global youth tobacco survey (GYTS) which is a school-based survey developed by WHO and US Centers for Disease Control and Prevention (CDC) in order to amplify the countries’ capacity for monitor consumption of tobacco among youth and provide operational guidance for the implementation and evaluation of tobacco prevention and control programs^23,24^. It is translated into Persian and being used by the Ministry of Health and Medical Education of Iran (MOHME) for preparing the GYTS country report for WHO^25^.

For the next phases, alongside with the other assessment tools mentioned in the following tables, researcher-made questionnaires will be employed for the assessment in the neighborhood and city phases.

### Measuring outcomes

Outcomes of each phase will be analyzed and compared using the pre-post method.

### Validity and reliability of tools

For the pilot phase, the validity and reliability of the tool are assured by the standardized test developed by WHO and being used by MOHME. In the next phases, the required tools will be made and the process for assuring validity and reliability for them will be administered.

The measuring tools for each phase were designed primarily using CDC^19^ and WHO^26^ guidelines, and preliminary details were identified. Each phase is a combination of different methods (including systematic observation, questionnaire, heuristic interview, and structured interview) due to abundant data and the need for accurate collection of data and the existence of different target groups.

When collecting data, the necessary steps are taken with each phase. In the next step, while providing a detailed description of the results, an analysis will be performed from different angles. The results are compared with the results of other phases, and finally, new strategies are provided to address the challenges. Since this research is context-based, it will be changeable according to the specific conditions of each region.

### Phases 1 & 2: Pilot and tobacco-free school

In the first phase (pilot phase), this project will be implemented in four schools of Varamin City; a series of measures will be carried out to sensitize and raise awareness and also to involve parents, educators, and students in selected schools (Table 1). In the second phase, all schools in Varamin City will be covered by the PAD project. Employing the experiences and lessons learned from the first phase, some other measures will be done for capacity-building, organizing, and engaging some of the volunteer students and empowering them as peer actors/educators regarding the changes (raising awareness on tobacco harms and prevention of tobacco use) and to develop it initially in their schools. Awareness-increasing will be carried out by educators and PAD-helpers through training sessions, face-to-face training by peers, and providing educational packages.

**Table 1:** Goals, Target groups, Stakeholders, projects, Measuring tools &Indicators of Pilot and tobacco-free school (Phases 1 & 2)

**Goals**	**Target groups**	**Stakeholders**	**Projects**	**Measuring tools**	**Indicators**
Prevention of tendency to tobacco smoking	Students	PAD-helpers Educators	Awareness-raising through educational packages	Questionnaire (WHO- GYTS)	Change in students’ literacy rates regarding tobacco
Protecting students from second-hand smoke	Students School staff Parents Other visitors to the school	Educators PAD-helpers School officials	Prohibiting smoking in all sections of the school Installation of anti-smoking signs	Checklist	Mentioning the prohibition of smoking in the disciplinary regulation Percentage of places that have the prohibition of smoking signs
Helping smokers quit	Students School staff Parents	Educators PAD-helpers	Introduction to IATA’s free center for smoking cessation in Varamin	Interview Questionnaire	The number of people who have quit smoking
Prohibition of the sale and use of tobacco products within the 100-meter radius of schools	Students School staff Parents Other visitors to the school Retailers around schools	Educators and PAD-helpers School officials Local councils Health houses	Installation of anti-smoking signs Prohibition of tobacco sale within the 100-meter radius of schools	Checklist	Percentage of places that have the prohibition of smoking signs percentage of sellers who have joined the project

he role of school officials is highlighted due to the need to include smoking prohibition laws in school disciplinary regulations. Restrictive laws apply not only to students, teachers, and school staff but also to all school visitors (such as parents and other guests).

Students, teachers, and school staff who smoke can benefit from IATA’s free smoking cessation clinic. At this stage, the effectiveness of the interventions will be assessed using interviews, questionnaires, and checklists.

### Phase 3: Tobacco-free neighborhood

The purpose of this phase is to preserve and enhance the achievements of the tobacco-free school phase and to expand them to the neighborhood level and to benefit all the residents of the neighborhood (Table 2). Preventing exposure to second-hand smoke and preventing the onset of tobacco use in adolescents and young people will be possible through the participation of all families and by PAD-helpers, educators, guilds, local councils, local trustees, and other governmental and non-governmental organizations active in the neighborhood.

**Table 2:** Goals, Target groups, Stakeholders, projects, Measuring tools &Indicators of Tobacco-free neighborhood (Phases 3)

**Goals**	**Target groups**	**Stakeholders**	**Methods**	**Measuring tools**	**Indicators**
Prevention of exposure to second-hand smoke Prevention of tobacco use onset	All families living in the neighborhood	PAD-helpers, mosques, local councils, other active institutions in the neighborhood, the mayors of districts, local trustees, health-care centers, local tradesmen	Awareness-increasing through educational packages Installation of anti-smoking signs Prohibition of selling single sticks of cigarettes and selling to people under 18 years of age	Checklist Questionnaire	Change in literacy rates regarding tobacco Percentage of places that have the prohibition of smoking signs percentage of sellers who have joined the project
Encouragement to change tobacco-related jobs	Guilds active in the field of tobacco	Related governmental institutions, PAD-helpers, guilds, mosques, health houses, local councils, other active institutions in the neighborhood	Awareness-increasing through educational packages Attracting the participation of relevant government agencies to support the change of jobs harmful to health	Checklist Interview	Percentage of businesses that have changed their jobs
Creating an appropriate space to quit smoking	All tobacco users in the neighborhood	Smoking cessation clinics	Introduction to IATA’s free center for smoking cessation in Varamin	Questionnaire	The number of people who have quit smoking

Increasing the awareness of active shopkeepers in the tobacco industry and consulting with relevant governmental agencies to provide the shopkeepers with some facilities are done to encourage and support the change of occupations harmful to health. Moreover, IATA’s smoking cessation clinic in Varamin will provide free services to consumers who are interested in quitting smoking.

At this phase, the effectiveness of the interventions will be measured using interviews, questionnaires, and checklists.

### Phase 4: Tobacco-free city

According to WHO, tobacco-free public places are a criterion for the realization of tobacco-free cities. Besides, it is necessary to pass laws and guidelines that guarantee its survival. Therefore, the target group in this phase is mainly officials and legislators who use the existing legal levers to protect citizens from the harms of tobacco (Table 3). Finally, the impact of the interventions will be measured by examining the level of law enforcement.

**Table 3:** Goals, Target groups, Stakeholders, projects, Measuring tools &Indicators of Tobacco-free city (Phases 4)

**Goals**	**Target groups**	**Stakeholders**	**Methods**	**Measuring tools**	**Indicators**
Improving the living standards of citizens Reducing long-term health care costs Reducing the damaging effects of tobacco on the environment	Officials and legislators Citizens	The mayor of Varamin, the Governor of Varamin, the heads of Education and Health-Care Administrations, NGOs	Preparation of anti-smoking laws and regulations Implementing a media campaign	Checklist	The rate of practical and legal changes in the city

Since the Comprehensive National Tobacco Control Law (ratified in 2006) has established measures to free public places from tobacco smoke, the development of intra-organizational guidelines and the provision of sufficient executive guarantees is a priority in this project.

The administrative-executive structure, actions, scheduling, and division in each of the phases are as follows.

**Figure 1:**
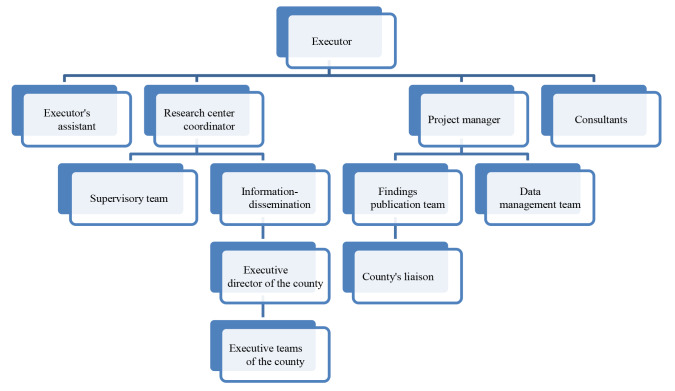
The administrative-executive structure of the project

The administrative-executive structure of the project: Initially, an administrative-executive structure was designed for the project, the components of shown in Figure 1.

### Ethical considerations

This project was reviewed by the Ethics Committee of the Tehran University of Medical Sciences and received the Code of Ethics IR.TUMS.DDRI.REC.1398.006. Also, to observe cultural considerations in this regard, the scope of the project was discussed through the evaluation and formulation of the principles of work, during initial meetings with the officials of the Office for Protection against Social Harms in the Ministry of Education of Varamin City.

### Safety considerations

To maintain the security of the participants in the project, especially in the neighborhood phase, the police and the governorate of Varamin, during the introductory meetings, became acquainted with the project and then provided the necessary permits. In areas where PAD-Helpers and the executive team are present in neighborhoods, IATA’s observers are associated with the team. Also, insuring the executors of the field research is the responsibility of IATA.

### Quality assurance

The measures are done to ensure the quality of this research are as follows: Providing the protocol before the start of the project; designing the managerial, executive, and monitoring structure of the project; documenting the process and recording the qualitative findings in the form of periodic reports at all phases of the project for reviewing by affiliated and independent experts; workgroup training of an executive team for the work in schools; and providing dedicated and distinct training manuals for both executors and stakeholders, in which the essential elements of the project are mentioned.

Other measures aimed at the improving the quality of the project included the recruitment and training of local forces, the localization of Global Youth Tobacco Survey (GYTS)^25^ questionnaire and other measurement and communication tools that fit increasingly into the multicultural, multiethnic, and religious context of the city of Varamin and lead to more participation of citizens.

## Discussion

In 1986, through the Ottawa Charter for Health Promotion, WHO emphasized the role of people’s participation in health promotion as a new perspective on global health and attracted the attention of policymakers and health planners around the world to this issue. Hence, promoting health through the definition of “empowering people to control the health-related factors” is selected as the pivot of WHO activities and health plans ^27^.

Following this new perspective, the emphasis on the individual and the particular role of therapists which was formerly underlying personal health and health psychology diminished, and communities and people’s participation turned into a topic of study and a new trend called community-based programs. In community-based programs, service and welfare processes and their related responsibilities are transferred to families and community members (structures), rather than the individual (agent and actor), so that community members have active participation at all stages of the program (needs assessment, prioritization, planning, implementation, and evaluation)^28^.

According to the WHO announcement on the framework of health-oriented urban planning, people have the right to be protected against the negative effects of secondhand smoke ^29^. This is one of the commitments under the Framework Convention on Tobacco Control (FCTC), and the members of FCTC, including Iran, should be committed to it. Therefore, due to the lack of a collaborative and effective program for preventing tobacco use from the younger ages, the current study was aimed at the prevention of tobacco use in adolescents, young people, and women, and through their participation, the realization of tobacco-free neighborhoods and cities.

This project seeks to achieve indicators of tobacco-free schools through direct and indirect education of students, parents, and educators, so that as the result of peer-to-peer and indirect education, achieving tobacco-free schools will also provide ground for achieving tobacco-free neighborhoods.

What caused the phases of the project starting from schools is the importance of tobacco prevention in the age in which most people begin to use tobacco. In the past few decades, there have been many activities in the world to prevent tobacco use in adolescents, in most of them schools have played a central role. Prevention of tobacco use by focusing on schools, the use of educational methods, and training of specific skills have had long-term persistent effects on smoking prevention^30^. Smoking in adolescence not only increases the likelihood of continuation of this behavior but smoking in the early years of life often results in increasing the risk of addiction. Those who start smoking in adolescence can hardly quit it compared to those who start it later ^31^.

The strengths of this project are not only the education of students and increasing their awareness, but also the creation and strengthening of social capital for participants, especially for students. To create an organized group of volunteer students as scouts and peer-to-peer educators, who will later participate in community-based activities, will lead to the creation of network and identity groups which are introduced in tobacco-related literature as factors for reducing risky behaviors such as tobacco use.

Since the participation of parents and educators is a vital factor in the success of the project, during the exploratory studies we have faced some barriers. One of these barriers was related to parents’ association meetings held periodically in schools. Introducing of PAD project to parents usually takes place during these meetings to create the least inconvenience to parents in terms of time. But some parents refused to attend the meetings, with the impression that these meetings are held with the aim of money collecting. The ineffective parents’ meetings will undermine approaches related to problem-solving and the processes of harm reduction. What can be learned from this experience is the negative impact of the process of privatization of schools and the educational system, which impedes the full participation of parents and school staff in solving the problems of students. In particular, solving social harms requires a process of trust and cooperation between the levels of individuals, families, and various institutions.

Another challenge to the project will be the general disagreement over the problem of introducing tobacco-related issues in schools. This controversy will lead some school principals to either refrain from participating in the project or have fewer partnerships. Besides, some parents might not be willing to participate in the project, with the impression that introducing and familiarizing students with tobacco can have adverse effects, and given the characteristics of adolescence period which is associated with restlessness and rebellion which may lead to the stimulation of students’ curiosity.

Another problem with the PAD project is the excessive focus on education and less attention to nurturing. In fact, due to the entrance exams for universities and the emphasis on formal grades and certificates, some school staff members opposed the allocation of part of school time to the introduction of tobacco-related issues. According to them, this may disrupt the process of education. Schools that have higher educational levels and higher scores in educational evaluation have less likelihood of participating in such projects. However, one of the most important functions of schools is the learning of social skills and abilities which is like vaccination of students at the time of entering the community.

Ideally, the desirable goal of this project is that schools can independently engage in activities such as the celebration of the World No-Tobacco Day, photo and painting exhibitions related to tobacco problems. Hence, through creating attractiveness in contests and group activities, this can provide an opportunity for constructive thought and decision-making regarding abstinence from tobacco use that can spread throughout the whole community, neighborhood and the entire city.

## Conclusion

Taking the initiative to create Non-smoking schools can be considered as an effective starting point in community-based projects to achieve a smoke-free neighborhood and a smoke-free city.

## Acknowledgements

We thank AliAta Taheri, Zahra Sadr and Fatemeh Matinkhah for their support, feedback and valuable suggestions.

## Conflict of interests

The Authors declare that there is no conflict of interests.

## Funding

This work was supported by Tobacco Control Research Center (Iranian Anti-Tobacco Association) [Grant Number: IATA/TCRC139612].

## Highlights

- School-based interventions are more effective if the school environment is also included.
- Restrictive laws may apply not only to students, teachers, and school staff but also to all school visitors (such as parents and other guests).
- Creating an organized group of volunteer students as scouts and peer-to-peer educators, who will later participate in community-based activities, will lead to the creation of network and identity groups as factors for reducing risky behaviors such as tobacco use.
